# Research progress of artificial intelligence in the early screening, diagnosis, precise treatment and prognosis prediction of three central gynecological malignancies

**DOI:** 10.3389/fonc.2025.1648407

**Published:** 2025-09-09

**Authors:** Qiyao Wang, Xinyu Zhou, Junying Wu, Weidong Miao, Boning Shen, Rui Shi

**Affiliations:** ^1^ Liaoning University of Traditional Chinese Medicine, Shenyang, Liaoning, China; ^2^ Key Laboratory of Ministry of Education for Traditional Chinese Medicine (TCM) Viscera-State Theory and Applications, Liaoning University of Traditional Chinese Medicine, Shenyang, Liaoning, China

**Keywords:** artificial intelligence, gynecologic oncology, medical imaging, early screen, diagnosis, treatment, prognosis, model

## Abstract

Early screening and diagnosis, precise treatment and prognosis prediction are pivotal for enhancing cancer patients’ survival rates and for improving their life quality, it is the same case in gynecological malignancies. Among gynecological malignancies, endometrial cancer, cervical cancer and ovarian cancer are regarded as the three major types due to their high incidence rates, clinical severity and unique biological characteristics. Nowadays, the early screening and diagnosis of gynecological malignancies mainly depends on imaging examinations, pathological evaluations, and serum biomarkers assessments, while they possess inherent limitations. The treatment and prognosis prediction of gynecological malignant tumors show significant individualized differences. Although the treatment methods have been continuously improved, there are still shortcomings such as complex drug resistance mechanisms that limit the treatment effect, the impact of treatment toxicity on the quality of life of patients, and the impact of varying doctor experience levels across hospitals on disparities in diagnosis and treatment quality. With the rapid evolution of artificial intelligence (AI), particularly through the integration of deep learning (DL) and machine learning (ML) algorithms, AI technologies have shown their benefits in medicine. AI technologies can efficiently analyze medical images, genomic data, and clinical information, thereby enable more precise diagnoses, facilitate the design of personalized treatment strategies, predict treatment outcomes and recurrence risks. AI has also been utilized in gynecological malignancies and exhibited substantial potential. This review summarizes the latest advancements of AI applications in the early screening, diagnosis, treatment and prognosis prediction of three central gynecological malignancies and dialectically discussed current limitations. It provides valuable insights into the future translational potential of AI in gynecological oncology.

## 1 Introduction

Gynecological malignancies, including cervical cancer (CCA), endometrial cancer (EC), and ovarian cancer (OC), pose a significant threat to women’s health (1). According to the statistics from the American Cancer Society, these three types of tumors have a high incidence and mortality rate worldwide and are one of the leading causes of cancer-related deaths among women (2). In 2022, there were approximately 1.473 million new cases and 680,000 deaths from gynecological malignancies globally (3), highlighting the severe challenge these cancers pose to women’s health worldwide. Currently, the diagnosis of gynecological malignancies mainly relies on imaging examinations (such as computed tomography (CT) and magnetic resonance imaging (MRI)), pathological analysis, and serum biomarker detection (4). However, these traditional methods face certain limitations in their practical applications. For instance, cytology tests (TCT) and human papillomavirus (HPV) detection in CCA screening have insufficient accuracy (with risks of false negatives and false positives) (5); the predictability of conventional diagnostic methods for EC and OC is poor (6, 7). These challenges have prompted the medical community to continuously explore new diagnostic and therapeutic approaches to improve the early detection rate and treatment outcomes of gynecological malignancies.

**Figure 1:**
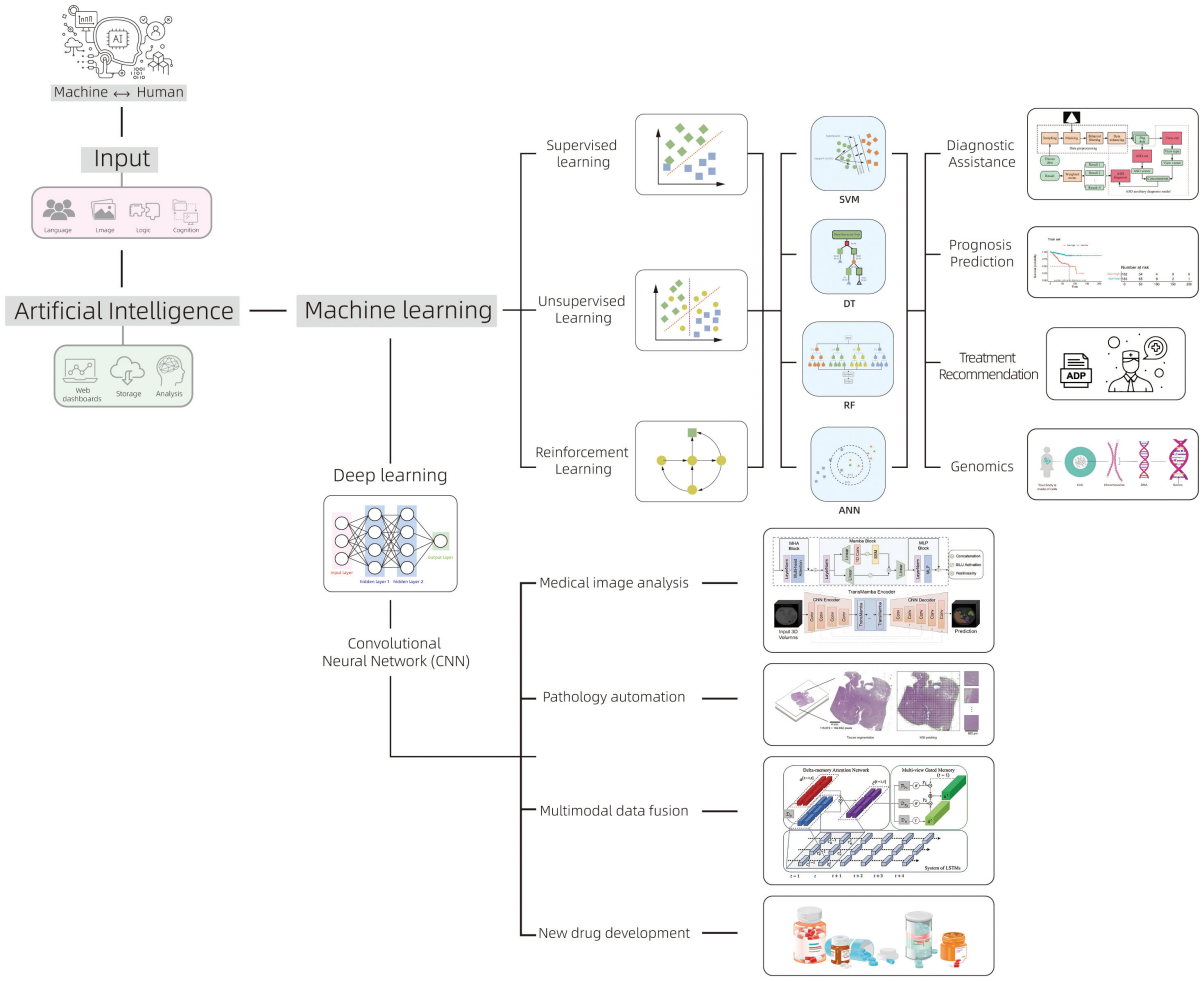
The classification of AI and its main applications in the field of oncology.

In recent years, AI, as an important branch of computer science, has widely permeated into multiple fields, demonstrating tremendous applicational potency in medicine. AI has achieved numerous breakthroughs in the early diagnosis of gynecological malignancies, personalized treatment plan design, and disease prognosis prediction benefiting from its significant advantages in handling massive data, pattern recognition, image analysis, and decision support (8). The core of AI technology is generally divided into two major categories: ML and DL (**Figure 1**). ML is a technology that can automatically extract patterns from large amounts of data. It builds models by analyzing historical data then makes predictions. These models can be continuously optimized and adjusted with new data input (9). ML can not only handle traditional data analysis tasks but also improve algorithms based on experience, gradually enhancing prediction accuracy and processing efficiency. Standard algorithms in ML technology include support vector machines (SVM), decision trees (DT), random forests (RF), and artificial neural networks (ANN) (10). DL is an important branch of ML. In recent years, with the improvement of computing power and the application of big data, DL has made significant progress. The basic theory of DL originates from the research of artificial neural networks, especially the development of convolutional neural networks (CNN), which enables DL to automatically extract deeper features and patterns from multi-level neural networks when dealing with complex data like medical images and genomic data (11). Unlike traditional ML algorithms, DL can automatically identify and abstract important information from data without the need for manual feature extraction design, which has led to its great success in image recognition, speech processing, and natural language processing et al. (12). The rapid development of DL continues to drive the advancement of AI, especially in medical image analysis. By training on large-scale datasets, DL can automatically detect, classify, and label lesion areas in images, significantly improves diagnostic efficiency and accuracy.

**Figure 2:**
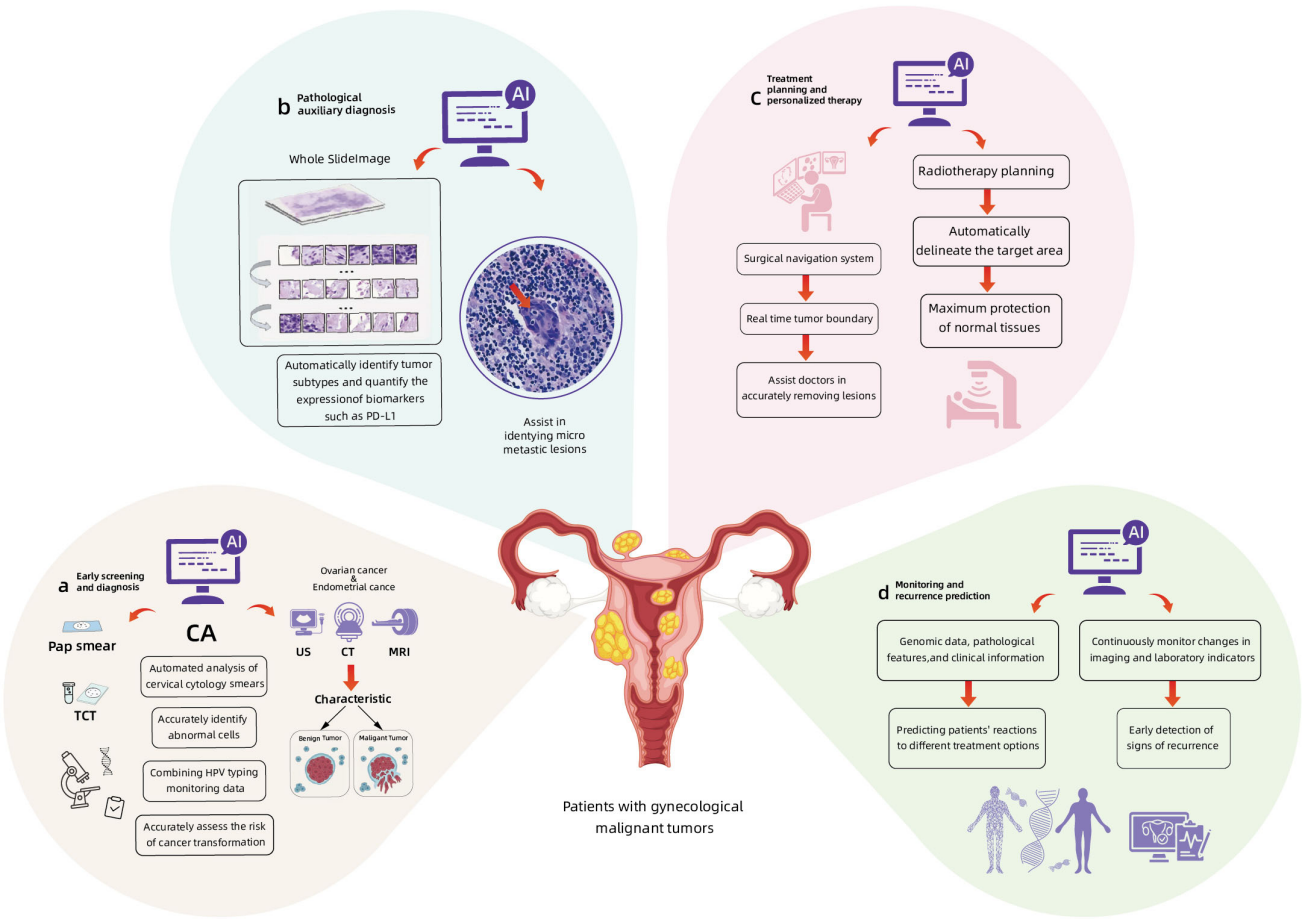
The applications of AI in the early screening and diagnosis **(a, b)**, treatment **(c)** and prognosis prediction **(d)** of gynecological malignant tumors.

The application of AI technology in imaging analysis, genomics, transcriptomics, proteomics, and microbiomics has been continuously deepened. Compared with traditional methods, AI has demonstrated multi-dimensional technical advantages in the diagnosis and treatment of gynecological malignancies (13). In terms of early screening and diagnosis, AI is capable of processing ultra-high-resolution pathological images and identifying microstructural features that are imperceptible to the human eye. In CCA, traditional cytological examinations, such as Pap smears, exhibit a 20% to 30% missed diagnosis rate due to the subjective interpretation by pathologists (14). In contrast, AI technology enables automated analysis of cervical cytology smears and accurate identification of abnormal cells, achieving a comparable accuracy rate to experienced pathologists (15). When integrated with HPV typing test data, AI can further enhance the precision of cancer risk assessment. For EC and OC, AI can effectively differentiate between benign and malignant tumors by analyzing imaging features from ultrasound, CT, and MRI scans, thereby significantly improves early diagnostic rate (16). In pathological diagnosis, digital pathology analysis systems based on DL can automatically identify tumor subtypes and quantify the expression of biomarkers such as PD-L1. In CCA and EC, AI can assist in identifying micrometastases. In terms of treatment decision support, AI can model complex nonlinear relationships, surpass the limitations of traditional statistical methods and demonstrate the unique advantages of AI. In surgical navigation systems, AI algorithms can identify tumor boundaries in real time, assists doctors precisely remove lesions. In radiotherapy planning, AI can not only automatically delineate target areas but also optimize dose distribution, maximumly protects normal tissues. Moreover, by integrating genomic data, pathological features and clinical information, AI can predict patients’ responses to different treatment regimens, providing a scientific basis for individualized treatment. In tumor prognosis management, AI detects early signs of recurrence by continuously monitoring imaging and laboratory indicators alterations, achieves more precise follow-up management. **Figure 2** summarizes the application of AI in the diagnosis, treatment and prognosis of gynecological malignant tumors. These technological advancements not only overcome the core limitations of traditional oncological medicine, such as insufficient diagnostic timeliness and homogenized treatment, but also reconstruct the precise diagnosis and treatment system for gynecological tumors through high-dimensional data fusion and dynamic monitoring.

Currently, AI researches in the field of gynecological oncology are progressing to a deeper and more sophisticated level. From morphological analysis based on medical imaging to the integration of multi-omics data (including radiomics, genomics, and proteomics), AI enables the development of more comprehensive models for early screening, diagnostic and therapeutic decision-making and prognosis prediction. Although challenges remain in areas such as data quality, algorithm interpretability, clinical translation, and standardized management, ongoing technological advancements and extensive clinical validations suggest that AI will become an indispensable intelligent assistance in gynecological oncology, propelling precision medicine to new heights. This review aims to explore the latest research progress of AI in the field of three major gynecological malignancies (CCA, EC, and OC), with a focus on analyzing the application achievements and challenges of AI in the early screening, precise diagnosis, clinical treatment, and prognosis prediction of these three major gynecological malignancies. It provides valuable references for the future transformation and application of AI technology in gynecological tumors, promots the in-depth development of AI in the research of gynecological malignancies, and ultimately provides more efficient and accurate diagnostic and therapeutic tools for clinicians, thereby improves the treatment effect and patients’ life quality.

## 2 The application of AI in CCA

CCA is the third most common malignant tumor among women worldwide and ranks first among female reproductive system tumors. Its occurrence is closely related to persistent HPV infection (17). According to the data from the World Health Organization (WHO), there are approximately 604,000 new cases and 342,000 deaths each year globally (18), with 90% of them occurring in low- and middle-income countries, where the incidence and mortality rates are relatively high. The natural course of CCA is divided into three stages: HPV infection, precancerous lesions (Cervical Intraepithelial Neoplasia), and invasive cancer. Screening and diagnosis are conducted through methods such as cervical cytology and HPV testing, colposcopy, cervical biopsy, imaging examinations, and serum tumor marker tests. Treatment options include surgery, radiotherapy, chemotherapy, targeted therapy, and immunotherapy, which are selected based on the patient’s age and fertility needs. In recent years, significant progress has been made in the diagnosis and treatment of CCA through AI (19). Through techniques such as image analysis, genomic data processing, and DL, AI is potential to improve clinical outcomes, increase the rate of early diagnosis, and enhance individualized treatment.

### 2.1 The application of AI in the early screening and diagnosis of CCA

Currently, CCA screening mainly relies on cervical smear and HPV testing. In some cases, colposcopy, CT or cervical biopsy and other techniques are needed for diagnosis (20). AI’s DL and image analysis technologies can automatically analyze cervical cell smears and pathological images, identify potential precancerous lesions and early cancer signs rapidly and improve the accuracy of screening and diagnosis. In 2020, Bao et al. (21) trained a supervised DL model based on 188,542 cervical cytology images and evaluated its performance through a multi-center clinical study. The area under the curve (AUC) of this AI system for detecting CIN2+ lesions was 0.762, with diagnostic rates of 92.6% and 96.1% for CIN2 and CIN3, respectively. Compared with skilled cytologists, AI-assisted reading showed a higher specificity (1.26 times); compared with cytopathologists, it is more sensitive and specified. Of note, in HPV-positive women, AI-assisted reading improved the specificity for detecting ≤CIN1 lesions while maintained the sensitivity. In the subsequent validation study (22), the scholar conducted AI statistical analysis on 703,103 colposcopy images (including normal and abnormal cases). The overall consistency rate between the AI model and manual cytology examination reached 94.7% (kappa = 0.92), and the detection sensitivity for CIN2+ lesions was 5.8% higher than manual reading. These results indicated that AI-assisted cytology can enhance screening accuracy, especially in the triage of high-risk populations, while maintaining diagnostic reliability comparable to experts. AI has demonstrated high accuracy in hierarchical diagnostics. In 2024, Wang and his team (23) developed and validated an Artificial Intelligence Cervical Cancer Screening System (AICCS) for cervical cytology classification, which integrated a dual-model architecture combining cell detection and whole-slide image classification. Trained and validated on a multicenter dataset of 16,056 cases, the AICCS achieved an accuracy of 89.2%, sensitivity of 94.6%, specificity of 89.0%, and AUC of 0.947 in prospective evaluations. Cytopathologists utilizing AICCS-assisted diagnosis demonstrated significant advantages across multiple metrics, with notable improvements in AUC, specificity, sensitivity and accuracy in comparisons to traditional manual interpretations. This system exhibited substantial potential of a standardized tool for cervical cancer screening. Nowadays, the interpretation of traditional cytology smears relies on manual observation, which is subject to strong subjectivity and low repeatability, affects the sensitivity of the test. To address this technical bottleneck, in 2021, Wentzensen et al. (24) developed a cloud-based whole-slide imaging system and trained a DL classifier for p16/Ki-67 dual-stained (DS) slides based on biopsy gold standards. This study included 4,253 patients and compared the diagnostic performance of AI-assisted DS with traditional Pap smears and manual DS. The results showed that the positive rate of AI-assisted DS was significantly lower than that of cytology and manual DS (P < 0.001), while maintained sensitivity, and its specificity was significantly better than Pap smears and manual DS (P < 0.001). Notably, AI-assisted DS reduced the colposcopy referral rate from 60.1% to 41.9% (P < 0.001). Therefore, this automated DS assessment system can eliminate the subjective differences in manual interpretation and ensure stable quality, maintain stable performance in both cytology and anal cytology tests. It not only reduces colposcopy but also performs well in a fully vaccinated population simulation, significantly improves the benefits of women’s health management.

The staging of CCA is according to the International Federation of Gynecology and Obstetrics (FIGO) criteria based on tumor size, depth of invasion and extent of spread, *etc.*, and is classified into four stages (25). In accordance with the pathological classifications published by WHO, CCA mainly includes squamous cell carcinoma (SCC), adenocarcinoma (AC), adenosquamous carcinoma and other types. In 2021, Wang et al. (26) developed a fully automatic DL-based diagnostic system for cervical lesions, which achieves rapid classification by analyzing whole-slide images (WSIs) of conventional Pap smears. In a test of 143 WSIs, the system demonstrated excellent diagnostic performance: accuracy of 0.93, recall of 0.90, F-score of 0.88, and Jaccard index of 0.84, with sensitivity comparable to the reference standard of pathologists. Fisher’s test confirmed (P < 0.0001) that the system significantly outperformed benchmark methods such as U-Net and SegNet in terms of accuracy, F-score, and Jaccard index. Moreover, processing a single WSI only takes 210 seconds, which is 19-20 times faster than the compared methods. This fully automatic system not only accurately identifies high-grade lesions requiring treatment but also has significant clinical application value due to its rapid processing capability and high operational efficiency. The prognoses of CCA varies among different histological subtypes. In 2022, Wang et al. (27) explored the application value of radiomics models based on multi-parameter MRI in differentiating histological subtypes of CCA. They retrospectively analyzed the preoperative pelvic MRI data of 96 patients with pathologically confirmed CCA (50 cases of SCC and 46 cases of AC), extracted 105 radiomics features from five sequences, and used clustering and logistic regression to analyze discriminatory efficacy. The texture heterogeneity of AC was significantly higher than that of SCC (P < 0.05). Among the sequences, T2SAG showed the best discriminatory efficacy (accuracy = 0.844, AUC = 0.86). The model combining the five sequences performed the best (AUC = 0.89, accuracy = 0.81, specificity = 0.94), significantly outperformed the single sequence. These results indicated that multi-parameter MRI radiomics could effectively distinguish the subtypes of CCA, among which AC shows more significant heterogeneity features, provided a new non-invasive classification method for clinical practice. In 2020, Miyagi et al. (28) developed a DL-based AI classification system that integrates colposcopic image features with HPV typing data for the precise classification of cervical squamous intraepithelial lesions (SILs). This study included 253 patients confirmed by biopsy, including 210 with high-grade squamous intraepithelial lesions (HSIL) and 43 with low-grade squamous intraepithelial lesions (LSIL). An AI model combining CNN and HPV feature tensors was constructed. This AI system demonstrated excellent diagnostic performance, with an overall accuracy rate of 0.941 (compared to 0.843 for doctors), a sensitivity of 0.956 and specificity of 0.833 for differentiating HSIL and LSIL (AUC = 0.963). In addition, the AI system performed exceptionally well in consideration of positive predictive value (0.977) and Youden’s index (0.789), confirming that multimodal AI can enhance the accuracy of cervical lesion classification and provide reliable assistance for clinical diagnosis. AI can also be used for the classification of cervical cells under uniform staining and different staining conditions. In 2021, Shi et al. (29) studied the clustered cell images of 966 Pap smear slides. They constructed a graph structure based on a new method of graph convolutional network (GCN), applied GCN to propagate node dependencies, and transformed cervical cell images into graph data structures for cluster analysis to reveal image relationships. They evaluated multiple indicators under both uniform staining and different staining conditions. The accuracy, sensitivity, specificity, and F-measure were 98.37%, 99.80%, 99.69%, and 0.998, respectively. The performance was superior to traditional methods, strongly verified that AI can be used for the automatic screening of cervical cytology.

### 2.2 Application of AI in the treatment process of CCA

Factors like tumor stage, the patient’s overall condition, and complications should take into account in the treatment of CCA. For early-stage CCA, surgery (such as total hysterectomy and lymph node dissection) is the preferred treatment. For locally advanced or recurrent cases, radiotherapy combined with chemotherapy (such as concurrent chemoradiotherapy with cisplatin) is adopted (30). For advanced cases, anti-angiogenic targeted drugs (such as bevacizumab) can be combined to enhance the therapeutic effect. In 2014, Kim et al. (31) conducted a case-control study involving 87 CCA patients to compare the therapeutic efficacies between robot-assisted surgery and traditional laparoscopic radical hysterectomy. The research findings demonstrated that the robot-assisted surgery group exhibited significantly superior outcomes in key indicators like intraoperative blood loss and postoperative recovery time, in comparison to the traditional laparoscopic group (P < 0.05). This study provides evidence-based medical support for the clinical application of robot-assisted surgery in CCA treatment, particularly in rapid recovery and reduced surgical complications. In 2018, Vizza et al. (32) conducted a comprehensive evaluation of the clinical efficacy of robotic single-site radical hysterectomy (RSSRH) combined with pelvic lymphadenectomy (PL) in patients with gynecological malignancies. This retrospective analysis encompassed 20 cases undergoing RSSRH with PL, with a focus on assessing surgical safety, postoperative recovery, complication rates, and the completeness of tumor resection. Results showed that the average operation time was 190 minutes, the median average intraoperative blood loss was 75 ml, and the median number of pelvic lymph nodes removed was 16 in this combined surgical approach. It indicates that single-site robotic surgery could minimize the invasion while maximize the precision and may become a new option for gynecological tumor surgery in the future. Since the onset ages of CCA are often young (33), and 7they often wish to preserve their fertility, robot-assisted laparoscopic surgery can be an ideal treatment option.

For CCA patients who are not suitable for surgery, radiotherapy is the best treatment option. In 2021, Mohammadi et al. (34) developed a deep convolutional neural network based on an improved ResU-Net architecture for the automatic delineation of organs at risk (OARs) in high-dose-rate brachytherapy (HDR-BT) for CCA. Through the analysis of imaging data from 113 patients with locally advanced CCA, the model demonstrated excellent performance, with Dice coefficients of 95.7 ± 3.7% for bladder, 96.6 ± 1.5% for rectum, and 92.2 ± 3.3% for sigmoid colon, and Hausdorff distances of 4.05 ± 5.17 mm, 1.96 ± 2.19 mm, and 3.15 ± 2.03 mm, respectively. The model achieved highly consistent results with manual delineation, significantly improved the repeatability and efficiency of radiotherapy planning. Similarly, in 2020, Zhang et al. (35) developed a DL-based 3D CNN architecture for the automated processing of CCA brachytherapy plans. Through the analysis of CT images from 91 patients, the study achieved precise segmentation of high-risk clinical target volumes and OARs, and innovatively used 3D skeletonization technology to complete the reconstruction of the applicator. Results showed that the model’s segmentation performance was significantly better than traditional methods, greatly improved the efficiency of treatment planning while ensured accuracy. In addition, in 2023, Wang et al. (36) proposed a 3D CNN model based on the attention gating mechanism (AG) to improve the automatic digitalization accuracy of interstitial needles in high-dose-rate brachytherapy for cholangiocarcinoma. By analyzing the CT treatment plan data of 56 cases from 17 cholangiocarcinoma patients, the performance differences among manual digitalization, traditional CNN, and CNN + AG were systematically compared. The model incorporated the attention mechanism demonstrated significant advantages, with a Dice similarity coefficient of 94%, a 5% improvement over the traditional CNN. The tip and shaft positioning errors were 1.1 mm and 1.8 mm, respectively, with an accuracy improvement of over 45%, and the HR-CTV D90 dose deviation was only 0.4%. This research effectively enhanced the model’s feature recognition ability for slender needle-like structures through an innovative attention gating design, which holds significant clinical application value and provides a reliable technical solution for the optimization of the automated process in brachytherapy.

### 2.3 Application of AI in the prognosis prediction of CCA

The prognosis prediction of CCA primarily depends on factors of cancer staging, tumor differentiation degree, and treatment modalities (37). Among these, the absolute number and ratio of metastatic lymph nodes constitute critical determinants influencing postoperative survival in CCA patients. In 2013, Chen et al. (38) investigated the impact of the absolute number of metastatic lymph nodes and the ratio of positive lymph nodes (RPL) on postoperative survival prognosis in patients. The study included 588 CCA patients who underwent radical hysterectomy and pelvic lymphadenectomy. Through cutoff survival analysis, the Kaplan-Meier method was employed to determine the RPL cutoff value at 10%, with corresponding 5-year survival rates (5-YSR) of 42.9% and 11.8%. The metastatic lymph node (MLN) cutoff values were set at 1 and 5, with 5-YSRs of 62.5%, 20.8%, and 7.8%, respectively. Furthermore, in the prognostic assessment of IA2 to IIA CCA patients post-radical hysterectomy and pelvic lymphadenectomy (RHPL), the hazard ratio for RPL was 3.195, which was higher than the MLN count hazard ratio of 1.578.In 2022, Liu et al. (39) developed a deep learning-based nomogram model (DLN) for predicting lymph node metastasis in CCA patients. Compared with the judgment of gynecologists, this model demonstrated higher sensitivity. In 2022, Xia et al. (40) developed a radiomics-nomogram-based predictive model, which demonstrated high AUC in both the training and testing cohorts. The accuracy, sensitivity, specificity, and AUC were 95.9% and 87.1%, 92.0% and 85.7%, 100.0% and 88.6%, and 0.988 and 0.922, respectively. This model provides a more precise basis for predicting preoperative pelvic lymph node metastasis in early CCA patients. In 2019, Tian et al. (41) demonstrated the potential of integrating HPV and human integrated genomic features with ML models in the risk stratification of cervical lesions. The average accuracy rate of the model’s prediction was 81.4%, providing a new tool for the early diagnosis of cervical lesions and being widely applied in the early screening and personalized treatment strategies of CCA. In 2021, Jajodia et al. (42) explored the combined use of radiomics features and apparent diffusion coefficient (ADC) to predict the prognosis of patients with sub-CCA after radiotherapy and chemotherapy through a supervised classification algorithm. The imaging data of 52 CCA patients who received radical radiotherapy and chemotherapy were retrospectively evaluated. The ADC values of the darkest parts of the tumors before and after radiotherapy and chemotherapy were calculated, and 851 radiomics features were extracted. Results showed that the model had good predictive performance for tumor recurrence and metastasis. The AUC of the model for predicting recurrence was 0.80, with a kappa value of 0.55, which was 40% higher in AUC and 223% higher in kappa value compared to using ADC alone; the AUC of the model for predicting metastasis was 0.84, with a kappa value of 0.65, with performance improvements of 2% and 140%, respectively. The study provided an effective method for stratifying CCA patients based on prognosis of predictive biomarkers.

## 3 Applications of AI in EC

EC is a group of epithelial malignancy that occur in the endometrium and is one of the most common tumors in women (43). According to the data from GLOBOCAN 2022, there are approximately 420,000 new cases and 97,000 deaths worldwide (17). The onset age range is mostly between 50 and 60 years old, and 90% of women with EC experience postmenopausal bleeding (44). EC is divided into four stages: early stage, locally advanced stage, regional spread stage, and distant metastasis stage (45). It is mainly screened through symptom assessment, imaging examinations, endometrial biopsy, etc., and the treatments include hysterectomy and lymph node dissection, but are prone to postoperative complications. At present, the diagnosis and screening of EC are confronted with challenges of high medical costs and a relatively high misdiagnosis rate, which directly affect the early detection of EC and the prognosis of patients. These are the key factors leading to the high mortality rate of EC. This situation highlights the significant value of AI in the field of EC diagnosis and treatment. Currently, the application of AI in EC diagnosis and treatment mainly focuses on two core areas: imaging diagnosis and clinical prediction. AI technology can enhance the accuracy of early EC diagnosis, achieve cost-effective risk stratification, and assist clinicians in better diagnosing and improving the prognosis of EC.

### 3.1 Application of AI in the early screening and diagnosis process of EC

The clinical manifestations of EC mainly include irregular vaginal bleeding, pelvic pain and masses, etc. Currently, clinical diagnosis mainly relies on imaging examinations and invasive pathological diagnosis (endometrial biopsy and curettage), but there are limitations like insufficient specificity and invasiveness (44). To optimize the diagnostic process, researchers are committed to integrating AI with multimodal data to develop more accurate molecular diagnostic strategies. Among them, transvaginal ultrasound (TVS), as the preferred screening method for postmenopausal bleeding patients, can provide important basis for the early identification of EC and precancerous lesions by evaluating the thickness and morphological characteristics of the endometrium (46). In 2024, Capasso et al. (47) developed a DL-based AI model to optimize the diagnostic performance of TVS for endometrial lesions. The study included 302 postmenopausal bleeding patients (including 153 cases of EC and 149 cases of endometrial atypical hyperplasia). The model was trained with a large amount of ultrasound image data to automatically identify the features of endometrial hyperplasia and malignant lesions. The model demonstrated good consistency in automatic segmentation (Dice coefficient = 0.79 ± 0.21), with AUC-ROC reaching 0.90 (95% CI: 0.88-0.92) in the validation set and 0.88 (95% CI:0.86-0.91) in the test set. The sensitivity and specificity remained at a high level of 0.86-0.87. This AI model has significantly improved the diagnostic accuracy of EC and atypical hyperplasia, with high sensitivity and specificity. Endometrial biopsy under hysteroscopic guidance is the gold standard for EC diagnosis. In 2021, Takahashi et al. (48) developed a DL-based hysteroscopic image analysis system for the automated diagnosis of EC. This study integrated three convolutional neural networks, Xception, MobileNetV2, and EfficientNetB0, to analyze hysteroscopic images from 177 patients (including 60 cases of normal endometrium, 21 cases of fibroids, 60 cases of polyps, 15 cases of atypical hyperplasia, and 21 cases of EC). The results showed that the diagnostic accuracy of the combined model significantly increased to 90.29% (80% for traditional methods), with a sensitivity of 91.66% (95%CI:77.53%-98.24%), a specificity of 89.36% (95% CI: 83.06%-93.92%), and an F value of 0.757. This innovative system achieves precise identification and classification of endometrial lesions. Compared with traditional standard methods, this model significantly improves the diagnostic accuracy to over 90%, provides a reliable automated solution for the early diagnosis of EC. In 2020, Troisi et al. (49) conducted a multicenter prospective study on 1,430 postmenopausal women with EC serum samples. They used high-performance liquid chromatography-mass spectrometry to quantitatively analyze the metabolites in the serum samples. Based on metabolomics, they employed multiple ML methods such as SVM and RF to conduct multi-dimensional statistical analysis of the patients’ serum metabolic indicators in accordance with the STARD reporting standard guidelines. The model’s accuracy in screening EC reached 99.86%, with both sensitivity and specificity exceeding 99%. This study confirmed that serum metabolomics combined with ML can serve as an effective supplementary means for the early screening of EC, especially for high-risk populations with atypical clinical symptoms.

According to the molecular typing standards of the Cancer Genome Atlas (TCGA) database, EC can be classified into four molecular subtypes with significant prognostic differences: POLE hypermutated type (DNA polymerase E), microsatellite instability high type (MSI-H), low copy number type (CNV-L), and high copy number type (CNV-H) (50). In 2021, Hong et al. (51) developed a DL-based computational pathology model for predicting the molecular subtypes of EC. This study employed the Panoptes multi-resolution DL architecture to analyze 456 formalin-fixed paraffin-embedded hematoxylin and eosin (H&E)-stained sections. The model achieved an AUC of 0.969 in differentiating endometrioid and serous subtypes, and the AUCs for predicting CNV-H, CNV-L, and MSI-H were 0.934, 0.889, and 0.827, respectively. Additionally, the model could accurately identify the characteristic gene mutations (TP53, PTEN, FAT1, and ZFHX3) of the CNV-H subtype, with prediction AUCs ranging from 0.781 to 0.873. This study confirmed that AI-assisted pathological image analysis can precisely predict the molecular subtypes of EC. In 2023, Xiong et al. (52) developed a DL-based computer-aided diagnosis system for predicting the depth of myometrial invasion in early EC. This study employed a multi-stage DL framework, integrating the SSD detection model and the Attention U-Net segmentation model to analyze MRI images of 154 EC patients. The system generated the uterine cavity line through an elliptical fitting algorithm to quantitatively assess the invasion depth, achieving an accuracy rate of 86.9% (sensitivity = 81.8%, specificity = 91.7%) in the classification of early EC. Notably, in the selection of the best MRI slice, the system’s accuracy rate reached 97.83%. Compared with traditional methods, this end-to-end prediction approach not only enhances diagnostic accuracy but also improves the interpretability of the results, thereby increased the precision of early EC diagnosis.

### 3.2 Application of AI in the treatment process of EC

Early-stage EC can be treated through total hysterectomy and lymph node dissection, etc. For locally advanced or high-risk patients, the combination of surgery with radiotherapy or chemotherapy can significantly improve the treatment outcome. With the continuous progress of minimally invasive techniques, robot-assisted surgery has shown significant advantages such as less trauma, rapid recovery, and less bleeding. It can significantly enhance the accuracy of surgery, reduce the incidence of complications, and effectively improve the patients’ postoperative life quality. It has gradually become one of the key technologies to improve the treatment effect of EC and reduce postoperative risks, especially in extensive lymph node dissection. In 2010, Cardenas-Goicoechea et al. (53) evaluated the value of robot-assisted surgery in the treatment of EC. A retrospective study included 275 EC patients who underwent robot-assisted surgery and traditional laparoscopic surgery at different time periods. The study found that robot-assisted surgery was comparable to traditional laparoscopic surgery in terms of key indicators of operation time and lymph node dissection, but the robot-assisted group resulted in significantly less blood loss (P < 0.05). Subsequently, the scholar expanded the research (54), conducting a survival analysis on 415 patients (183 in the robotic group and 232 in the laparoscopic group), and re-evaluated the differences in long-term survival rates and tumor recurrence rates between the two surgical methods. There was no statistically significant difference in the 3-year overall survival rate (93.3% vs 93.6%) and disease-free survival rate (83.3% vs 88.4%) (P > 0.05), the recurrence rates were also similar (14.8% vs 12.1%). These studies confirmed that robot-assisted surgery, while ensuring oncological safety, shows the advantage of minimally invasion and can be a reasonable choice for the surgical treatment of EC. In 2022, Argenta et al. (55) compared the efficacy of robot-assisted surgery (RAS), laparoscopic surgery (LS), and open surgery (OS) in the treatment of stage IA ECs. The study included 175 patients from 2010 to 2022, with 55 in the RAS group, 40 in the LS group, and 80 in the OS group. The results showed that the RAS group had the shortest operation time (P < 0.05), and both the RAS and LS groups had significantly less intraoperative blood loss in comparison to the OS group (P < 0.05). There were no statistically significant differences in progression-free survival and overall survival among the three groups for stage IA patients. Notably, the RAS group showed the lowest rate of perioperative complications (Clavien-Dindo ≥ 1, P = 0.02), and the grades of occurred complications were all < 2. The learning curve analysis indicated that the operation time of the RAS group was significantly shortened after completing 10 cases (regardless of whether pelvic lymph node dissection was performed). The study demonstrated that RAS is a safe and effective surgical approach for stage IA EC, with less trauma and fewer complications.

For elderly female EC patients, traditional OS poses surgical risks, while RAS offers a safer minimally invasive option. In 2010, Lowe et al. (56) retrospectively analyzed the application and perioperative outcomes of RAS in 395 elderly EC patients aged 80-95 years. The study found that the application of RAS did not increase surgical risks, with a median hospital stay of only one day (80% of patients were discharged within two days after surgery), and 96% of elderly patients successfully completed RAS. The overall complication rate was 7.4%. It proved that the RAS is safe and effective for elderly EC patients, its minimal invasive characteristics can reduce postoperative recovery time and complications, making it a feasible and selectable treatment option, breaking the age limit on surgical methods. Furthermore, in 2018, Peng et al. (57) conducted a study on 497 patients who underwent gynecological laparoscopic surgery, including 209 obese/morbidly obese patients. They showed that although obesity increases the difficulty of surgery, laparoscopic surgery is still safe and feasible. The intraoperative complication rate in the obese group was 9-11%, and the postoperative complication rate was only 2.41%. The average hospital stay was significantly shortened (P < 0.05), and the readmission rate was as low as 2.13%. This study highlights the potential clinical utility of artificial intelligence-assisted surgical techniques in defined patient populations.

### 3.3 Application of AI in the prognosis prediction of EC

The prognosis assessment of EC has a significant impact on the survival rate of patients. However, traditional clinical methods come with limitations in predicting disease progression and individualized treatment plans. In 2022, Bhardwaj et al. (58) summarized the application effects of various ML models when combining clinical, pathological, and imaging data, verified that ML can enhance the accuracy of predictions by extracting complex data features. In prognosis prediction, they proposed the MEDomics method, which integrates electronic health records (EHR) with a continuous learning infrastructure into an AI framework. It can effectively handle multimodal clinical data containing thousands of cancer patients and millions of data points, improves the accuracy of early diagnosis, risk assessment, and treatment outcome prediction of EC, especially in the screening of high-risk patients. It helps overcome the limitations of traditional clinical methods, particularly in dealing with complex high-dimensional datasets such as clinical, pathological, imaging, and genomic data. Variety of ML algorithms have been widely applied in the treatment and prognosis of cancer. In 2020, Chen et al. (59) analyzed the immune-related genes in the tumor microenvironment of 521 EC patients and developed a tool called ESTIMATE for predicting tumor content and the infiltration degree of stromal and immune cells. They systematically analyzed the expression patterns of immune-related genes using multiple public databases (such as TCGA), screened out genes related to the tumor immune microenvironment (TME), and evaluated their correlation with patient survival, further verified the prognostic value of immune-related genes. Currently, this method has been widely applied in determining the immune and stromal scores of various cancers, like breast cancer (60), and colon cancer (61). ESTIMATE can easily and effectively estimate the proportion of tumor cells in samples. By incorporating endothelial cell features and tumor type-specific normal epithelial cells, the system scores are highly correlated with clinical tumor samples and tumor cell purity, provides strong support for the molecular feature analysis and personalized treatment of tumors. Accurate determination of whether EC invades the myometrium and the depth of invasion is crucial for treatment decisions. In 2020, Chen et al. (62) developed a DL-based MRI image analysis system to assess the degree of myometrial invasion of EC. This study included T2WI data from 530 patients (99 cases of deep myometrial invasion and 431 cases of superficial invasion), and trained the prediction model using sagittal and coronal images. The model’s accuracy in identifying lesion areas reached 77.14%-86.67%, and it performed well in judging the depth of myometrial invasion (sensitivity: 66.6%, specificity: 87.5%, accuracy: 84.8%), with a particularly outstanding negative predictive value (94.6%). Compared with the routine diagnosis of radiologists, this AI system demonstrated significant advantages and could assist clinicians to improve diagnostic efficiency and reduce human error.

Lymph node metastasis is one of the main ways of EC metastasis, it often indicates the deterioration of the condition and the complication of treatment. In 2019, Gunukan et al. (63) developed a prediction model based on ML and Naive Bayes algorithms to assess the risk of lymph node metastasis in EC patients. This study included 762 patients and comprehensively analyzed key pathological features such as FIGO stage, depth of myometrial invasion, and cervical stromal invasion. The model’s prediction accuracy was as high as 84.2%-97.6%, provides a reliable basis for the clinical formulation of individualized treatment plans. The research confirmed that the precise assessment of the depth and extent of myometrial invasion is of significant guiding value for treatment decisions. In 2024, Volinsky-Fremond et al. (64) developed a multimodal DL model named HECTOR for predicting the recurrence risk of EC. This study integrated H&E stained whole-slide images and clinical data from 2072 patients with stage I-III EC to construct an ensemble algorithm with a three-arm architecture. The model was compared with traditional clinical prediction models using five-fold cross-validation. The C-index scores in the test set were 0.789, 0.828, and 0.815, outperformed the current gold standard of traditional pathology and other prediction models. Notably, the prediction accuracy for the low-risk group’s 10-year distant recurrence-free survival reached 97%, demonstrated a significant advantage and facilitated early intervention and more precise treatment planning. AI can also perform proteomic analysis by analyzing cervical-vaginal fluid and plasma samples to identify protein features associated with EC. In 2024, Njoku et al. (65) conducted proteomic analysis using blood and cervicovaginal fluid samples from 118 postmenopausal bleeding patients (53 cases of EC and 65 controls), and applied the Boruta algorithm to screen out key protein markers (including HPT, LG3BP, etc.) and constructed a RF prediction model. This model demonstrated excellent detection performance for EC, with an overall AUC of 0.91-0.98 (sensitivity: 83%-98%, specificity: 78%-95%). The study also identified stage-specific marker combinations: early stage (CNDP1, AUC: 0.82-0.95), stage I (HPT, AUC: 0.87-0.97), and advanced stage (APOE, AUC: 0.92-1.00). This research, based on the natural shedding characteristics of endometrial tumors, demonstrated the feasibility of detecting EC through cervicovaginal fluid, and further validation in larger independent cohorts is needed.

## 4 The application of AI in OC

OC is the fourth most common gynecological tumor worldwide and ranks third in mortality rate among gynecological tumors. It mainly occurs in the epithelial tissues, sex cords, or ovarian stromal cells (66). Due to the ovaries are deep in the abdominal cavity, the early symptoms of OC are not obvious, and over 70% of cases are not detected until the advanced stages (III-IV), while the treatment becomes extremely difficult and survival rates are low. Therefore, OC is often regarded as a “silent cancer” (67, 68). OC is a complex and highly heterogeneous malignancy and its growth and metastasis are regulated by multiple mechanisms. Tumor cells obtain nutritional supply through abnormal angiogenesis, promots their expansion and metastasis (69, 70); meanwhile, the immunosuppression in the tumor microenvironment and the role of non-coding RNAs (71, 72) exacerbate immune evasion and treatment resistance, making the diagnosis, treatment, and prognosis assessment of OC face significant challenges. Especially, the characteristics of metastasis and tumor recurrence further increase the difficulty of treatment. Traditional diagnostic and therapeutic methods often struggle to cope with these complexities. Imaging and genetic testing methods are complex, although providing certain information, could not fully reveal the tumor dynamics and meet personalized patients’ needs. Therefore, OC faces issues such as insufficient efficacy, drug resistance, and recurrence, alternative efficient and precise diagnostic tools are desperately needed. AI technology demonstrates distinct advantages in processing large-scale and high-dimensional medical data. Its application in the diagnosis and treatment of OC are potential of transforming traditional clinical paradigms and offering enhanced support for personalized treatment strategies and precision medicine (73).

### 4.1 The application of AI in the early screening and diagnosis of OC

Early screening and diagnosis techniques are important in OC. Different types of ovarian tumors need different management and treatment plans (74). Therefore, accurately identifying whether an ovarian tumor is benign or malignant is of vital importance. AI, especially DL algorithms, have been proven to effectively analyze imaging data such as ultrasound(US), CT, MRI, and positron emission tomography(PET) to assist clinicians in accurate identification, thereby enable the early diagnosis and classification of OC (75, 76). In 2022, Saida et al. (77) compared the performance of DL and radiologists in diagnosing OC using MRI. Data from 194 patients with pathologically confirmed OC or borderline tumors and 271 patients with non-malignant lesions who underwent MRI were collected. The researchers analyzed T2WI, DWI, ADC maps, and fat-suppressed contrast-enhanced T1WI, trained and tested the model on 1,798 images. The sensitivity, specificity, accuracy, and AUC of the model were comparable to those of radiologists. Among them, CNN demonstrated the highest diagnostic performance in ADC maps (specificity = 0.85; sensitivity = 0.77; accuracy = 0.81; AUC = 0.89). CNN demonstrated superior diagnostic performance compared to radiologists in MRI diagnosis of OC, particularly in the ADC map sequence. In 2022, Gao et al. (78) developed a deep convolutional neural network (DCNN) model for the automated assessment of US images to enhance the accuracy of OC diagnosis. A total of 3,755 pelvic US images from patients were included in this study. It was found that the AUC of DCNN in the internal validation set reached 0.911, and in the two external validation sets, it was 0.870 and 0.831, respectively. The diagnostic accuracy (81.1%-88.8%) was higher than that of 35 radiologists. Notably, with the assistance of DCNN, the diagnostic accuracy of six radiologists increased to 0.876, representing a significant improvement (P < 0.05). This indicated that the performance of DCNN exceeded radiologists and was even comparable to the expert level, potentially enhanced the diagnostic accuracy.

With the rapid development of AI technology, an increasing number of researchers have begun to attempt to enhance the diagnostic accuracy of OC by integrating multiple data sources, including imaging data, clinical information, pathological data, and genomic data, etc. In 2024, Xiang et al. (16) developed an interpretable AI model named OvcaFinder, which was constructed by integrating DL predictions of US images, radiologists’ Ovarian-Adnexal Reporting and Data System (OARDS) scores, and conventional clinical variables. The AUC of OvcaFinder was 0.978 in the internal test set and 0.947 in the external test set, outperformed traditional clinical and DL models. The auxiliary use of this model significantly improved the AUC values and reading consistency of radiologists, with false positive rates reduced by 13.4% and 8.3% respectively, indicating that OvcaFinder can significantly enhance the accuracy and consistency of OC diagnosis. Radiomics is a field that extracts quantitative features (such as texture, shape, intensity) from medical images (CT, MRI, PET) through high-throughput methods, and combines AI for analysis, converting image data into a high-dimensional feature library that can be mined (79). In 2021, Li et al. (74) developed a 2D radiomics method combined with CT technology to distinguish benign and malignant ovarian tumors. The study included CT images of 134 patients with ovarian tumors. The maximum diameter lesion area was identified using ITK-SNAP software, and texture features were extracted and selected through the maximum relevance, minimum redundancy, least absolute shrinkage and selection operator (LASSO) algorithm. The radiomics features demonstrated stable discrimination ability in both the training set (AUC = 0.88) and the test set (AUC = 0.87), while the nomogram integrating clinical parameters showed better predictive performance (training set AUC = 0.95, test set AUC = 0.96). External validation further confirmed the generalization ability of the model (AUC = 0.83-0.95). Decision curve analysis (DCA) indicated that the nomogram had good clinical utility. The study model showed high diagnostic efficiency and was helpful for the prediction of benign and malignant ovarian tumors. In 2023, Wang et al. (80) developed a DL-based automated segmentation tool for CT image analysis of OC. The study included data from 367 patients and compared the segmentation performance of four model architectures, found that the 3D U-Net cascade architecture performed the best, with a median Dice score of 0.941 (Jaccard index = 0.890, sensitivity = 0.973, and precision = 0.925). The analysis showed a high consistency between the automated and manual segmentation of tumor volume, and 85% of the radiomics features remained stable. This study confirmed that DL can achieve precise automated segmentation of OC and has the potential for clinical batch application.

OC is mainly divided into epithelial ovarian cancer (EOC), non-epithelial OC and borderline ovarian tumors (BOT) (81). Among them, EOC is the most common type, accounting for more than 90% of OC cases, and is further classified into type I and type II (82). Accurately distinguishing different types of OC is of great significance for treatment plans and prognosis assessment. In 2023, Wang et al. (83) developed a DL model based on conventional MRI to distinguish BOT from EOC. A total of 201 patients (102 BOT and 99 EOC) were collected, and T1WI/T2WI images were used to train the deep supervised U-net^++^ model. In the test set, the DSC of sagittal T2WI was 0.73 ± 0.25, that of coronal T2WI was 0.76 ± 0.18, and that of axial T1WI was 0.60 ± 0.24. The AUC of the model based on T2WI for differentiating BOT from EOC was 0.87 (accuracy = 83.7%, sensitivity = 75.0%, specificity = 87.5%), which was significantly better than the 0.75 AUC of radiologists (accuracy = 75.5%, sensitivity = 96.0%, specificity = 54.2%, P < 0.001). This DL model demonstrated superior performance in the differential diagnosis of ovarian tumors and could accurately distinguish benign ovarian tumors from OC. In 2022, Xu et al. (84) developed a radiomics classification model for epithelial ovarian tumors (EOTs) based on DWI and ADC maps. The study included 146 EOT patients (34 with BOT, 30 with Type I and 82 with Type II EOT). Key radiomics features were selected from 390 features using the LASSO algorithm and combined with clinical parameters to establish a nomogram. Validation showed that both the radiomics model (AUC = 0.915) and the nomogram (AUC = 0.954) were significantly superior to the clinical model (AUC = 0.852) in differentiating BOT from malignant EOTs. For the classification of Type I/II, the AUC of the radiomics model reached 0.905 (0.735 for the clinical model). DCA and net reclassification index analysis confirmed that the combined model showed the best clinical net benefit, provided a reliable quantitative imaging tool for the early classification of EOTs. Furthermore, AI played a pivotal role in histopathology, assisted pathologists in interpreting tissue samples. In 2023, Breen et al. (85) conducted a systematic review on the application of AI in OC histopathology, demonstrated that AI algorithms can accurately classify tumor subtypes, assess tissue heterogeneity, and detect microvascular invasion. This advancement not only enhances diagnostic accuracy but also minimizes human errors, ensures clinicians obtain more rapid and reliable results.

### 4.2 The application of AI in the treatment of OC

In recent years, AI technology has demonstrated revolutionary potential in the field of precise treatment for OC. In 2022, Wang et al. (86) developed a weakly supervised DL method, the AIM2-DL model, to guide OC treatment and identify effective biomarkers. A database was established containing immunohistochemical tissue samples of AIM2, C3, C5, and NLRP3 from patients diagnosed with EOC and primary peritoneal cancer (PSPC) who received bevacizumab treatment. In the first experiment (with 66% of the data as the training set and 34% as the test set), the model achieved high accuracy of 0.92, recall of 0.97, F-score of 0.93, and AUC of 0.97. In the second experiment using five-fold cross-validation, it achieved high accuracy of 0.86 ± 0.07, precision of 0.9 ± 0.07, recall of 0.85 ± 0.06, F-score of 0.87 ± 0.06, and AUC of 0.91 ± 0.05. Kaplan-Meier progression-free survival (PFS) analysis and Cox proportional hazards model analysis further confirmed that the AIM2-DL model can distinguish patients with low cancer recurrence after treatment from those with disease progression, which can be used to develop customized and personalized treatment methods for EOC and PSPC patients. In 2024, Leng et al. (87) developed and validated a radiomics model based on CT images for assessing the preoperative metastasis risk of EOC. The study analyzed 109 EOC patients, extracted 851 radiomics features from preoperative enhanced CT images, and selected 11 key features through the LASSO algorithm. Based on these features, the researchers constructed clinical models, radiomics models, and combined models, evaluated their performances through ROC curves and DCA. The study found that 75 patients (68.8%) had metastasis. The AUC of the radiomics model in the training cohort was 0.929 (95% CI: 0.8593-0.9996), and in the test cohort was 0.909 (95% CI: 0.7921-1.0000). By combining clinical features with radiomics scores, the researchers also constructed a radiomics nomogram. DCA analysis indicated that when the threshold probability exceeded 15%, the nomogram demonstrated the best net benefit. Compared with the clinical and radiomics models, the nomogram had a significant advantage in the diagnostic performance for evaluating metastasis of OC, provided effective support for personalized clinical treatment.

### 4.3 Application of AI in the prognosis prediction of OC

Monitoring the treatment response of OC patients is crucial for evaluating the efficacy of chemotherapy and other therapies. In 2022, Wang et al. (88) proposed a weakly supervised DL method that accurately predicts the effect of bevacizumab treatment in OC patients by analyzing whole-slide images of histopathological H&E staining, without requiring any local annotation regions provided by pathologists. This method demonstrated high accuracy (0.882 ± 0.06), precision (0.921 ± 0.04), recall (0.912 ± 0.03), and F-score (0.917 ± 0.07) in a 5-fold cross-validation, outperformed the latest DL methods previously reported. On an independent TMA test set, the three methods achieved high recall rates of 0.946, 0.893, and 0.964, respectively. Statistical analysis revealed that patients predicted as non-responsive had a significantly higher risk of cancer recurrence (hazard ratio = 13.727) compared to those predicted as responsive. These results suggested that this method can help to identify patients without positive responses, avoid unnecessary treatments, while provide treatment guidance and recurrence risk prediction for OC patients. Platinum-based chemotherapy is the cornerstone of treating high-grade serous ovarian cancer (HGSOC), but choosing the appropriate treatment plan depends on the patient’s response to chemotherapy. Currently, there is no biomarker that can rapidly predict the response to platinum-based therapy. Therefore, in 2024, Ahn et al. (89) developed a pathological risk classifier based on histopathological images, PathoRiCH, to predict the treatment response to platinum-based drugs in patients with HGSOC. PathoRiCH was trained on an internal cohort (n = 394) and validated on two external cohorts (n = 284 and n = 136), demonstrated that it could significantly distinguish the platinum-free interval between favorable and unfavorable response groups. By combining molecular biomarkers, PathoRiCH enhanced the accuracy of risk stratification and explained the decision-making process through visualization and transcriptome analysis. Thus, the predictive performance of this DL model is superior to current molecular biomarkers, provides an effective solution for personalized treatment strategies in high-risk gynecological tumors.

Accurate prognosis prediction is an important component of personalized treatment strategies for OC. Nowadays, there are relatively few reported prognostic biomarkers. In 2019, Wang et al. (90) proposed a DL method to extract prognostic biomarkers for HGSOC from preoperative CT images and developed a non-invasive recurrence prediction model. This study recruited 245 patients and trained the DL network with 8,917 CT images from 102 patients to extract prognostic biomarkers. Combined with Cox proportional hazards regression, the DL-CPH model was developed to predict individual recurrence risk and 3-year recurrence probability. The results showed that in the validation cohort, the concordance index of the DL-CPH model was 0.713 and 0.694, respectively, and Kaplan-Meier analysis distinguished the high-risk and low-risk groups for recurrence. The AUC for 3-year recurrence prediction was 0.772 and 0.825, respectively, demonstrated good calibration and DCA performance. Compared with clinical features, DL features demonstrated stronger prognostic value. Therefore, this DL method provides a preoperative non-invasive individualized recurrence prediction model for HGSOC, which is of great prognostic value in tumor recurrence prediction and can extract effective prognostic biomarkers from CT data without follow-up. In 2020, Bao et al. (91) developed a novel prognostic predictive model by integrating OC data from the GEO and TCGA databases. The study identified differentially expressed genes significantly enriched in the G2/M checkpoint pathway and constructed a multi-gene signature using LASSO Cox regression. The model was validated by time-dependent ROC curves (AUC = 0.72) and could effectively distinguish high-risk from low-risk patients (P < 0.001). Further research identified SNRPD1 and EFNA5 as core prognostic markers, with high expression of EFNA5 and low expression of SNRPD1 both indicated a poor prognosis. WGCNA analysis revealed the correlation between gene features and clinical pathological parameters, and simultaneously identified 16 potential targeted therapeutic drugs. A new gene signature was established as an independent prognostic factor to assess the prognosis risk of OC patients and promote the implementation of personalized treatment. Complete cytoreductive surgery is the most important prognostic indicator for EOC. In 2022, Laios et al. (92) developed an interpretable AI model based on the XGBoost algorithm to predict the possibility of complete cytoreductive surgery (R0 resection) in patients with advanced EOC. This study retrospectively analyzed the data of 571 patients who underwent cytoreductive surgery, integrated clinical and surgical-related parameters to construct a predictive model. The model demonstrated excellent predictive performance (AUC = 0.866, 95% CI = 0.8-0.93). Through Shapley Additive Explanations analysis, the study identified key predictive factors of the OC score, peritoneal cancer index, surgical complexity score, patient age, and tumor volume. Research has confirmed that R0 resection is the most important independent prognostic factor for EOC. This model comes with clinically applicable predictive accuracy, its explainable features further facilitate surgical quality assessment and clinical decision-making. Multiple studies have shown that AI has played a role in predicting PFS in patients with advanced OC (93, 94). In 2021, Wang and Lu (95) developed a predictive model based on PET and CT radiomics features for evaluating the PFS of advanced HGSOC. This study retrospectively analyzed multimodal imaging data from 146 patients with epithelial ovarian tumors (including 34 BEOT, 30 type I EOC, and 82 type II EOC). The predictive model was established by screening features through LASSO regression and combining them with multivariate logistic regression, and a nomogram integrating radiomics features and clinical parameters was also constructed. The validation results showed that the radiomics model was significantly superior to the traditional clinical model in differentiating benign and malignant lesions (AUC = 0.915 vs 0.852) and distinguishing type I/II cancers (AUC = 0.905 vs 0.735). DCA confirmed that this model provided a higher clinical net benefit. This multimodal radiomics approach provides an effective quantitative tool for precise prognostic assessment of HGSOC. In 2025, Ma et al. (96) developed a new DL framework that used preoperative laparoscopic images to evaluate the clinical outcome after treatment and assess short-term and long-term PFS. This DL can accurately stratify HGSOC patients based on laparoscopic images, thereby optimized early treatment plans. In 2024, Zhang et al. (97) developed an AI-based drug sensitivity prediction system to optimize the precise treatment strategy for OC. The study analyzed the drug response characteristics of 21,937 single cells using the Beyondcell algorithm and identified four patient subgroups with significant treatment differences by combining TCGA multi-omics data (among which the CS subtype showed broad-spectrum drug sensitivity). They innovatively constructed a DL prognostic model with a KAN architecture and evaluated its performance through three external validation sets in the GEO database. Research has found that endothelial cells are resistant to paclitaxel, doxorubicin and docetaxel, suggesting that they are potential targets. Through cluster analysis, four patient subtypes were identified, among which subtype carbon disulfide was most sensitive to all drugs. By using the KAN architecture to replace the traditional model, the performance of prognosis prediction was significantly improved.

## 5 Threats to validity and limitations of AI in the application of gynecological malignant tumors

The application of AI in gynecological oncology has demonstrated promising results, but the validity of existing research is limited by several methodological challenges, particularly selection bias in studies and threats to internal validity. A critical assessment of these limitations is crucial for guiding future research and clinical translation.

### 5.1 Selection bias

#### 5.1.1 Insufficient representation of demographic and clinical characteristics

If AI models are trained on non-representative data (e.g., only certain demographic characteristics, tumor subtypes, or imaging modalities), they may perform poorly in real-world applications (98, 99). For instance, Esteva et al. (100) introduced a deep neural network model capable of classifying skin cancer at the level of dermatologists. The study used a large number of skin lesion images for training, but subsequent research pointed out that the model lacked representation of darker-skinned populations (such as African and Asian) in the training data, resulting in significant lower diagnostic accuracy in these groups compared to the Caucasian population. This disparity may stem from the differences in the presentation of skin lesions among different races and the racial imbalance in the training data. Therefore, more diverse datasets should be incorporated in the development of medical AI.

#### 5.1.2 Inconsistent annotation

The training of AI models requires a large amount of high-quality annotated data. However, the annotation of these clinical training sets usually relies on manual identification and cancer classification by doctors. The subjective differences among different doctors in the diagnosis and classification process may lead to inconsistent annotations, thereby affecting the accuracy and generalization ability of the model. For example, the inconsistency among pathologists in EC grading may propagate bias into the AI training labels. Moreover, if there are incorrect labels in the training data (such as mislabeling benign lesions as malignant), the model may learn incorrect patterns, leading to clinical misdiagnosis (101).

#### 5.1.3 Time and concept drift

Over time, the continuous advancement of medical imaging technology or diagnostic criteria may render AI models obsolete. Concept drift refers to the change in the underlying relationship between training data and new data, leading to a decline in model performance. For instance, after 2020, certain patchy ground-glass density patterns in chest X-rays may no longer be labeled as bacterial pneumonia but as COVID-19 pneumonia (102).

### 5.2 Internal validity

#### 5.2.1 Confounding variables

Unconsidered factors (such as comorbidities, hormone therapy, or imaging artifacts) may distort the predictions of AI. Patients with endometriosis may show imaging features that AI might misinterpret as malignant. For instance, when Dong et al. (103) developed an AI model to assess the depth of myometrial invasion in early EC, it showed a significant misclassification rate for patients with uterine fibroids or endometrial polyps, suggesting that current AI systems still need to improve their ability to distinguish complex coexisting pathologies.

#### 5.2.2 Overly simplified evaluation

Current evaluation methodologies may place excessive emphasis on overall performance metrics (e.g., AUC), often neglecting critical aspects such as subgroup analyses (e.g., stratification by disease stage) and assessments of clinical utility (e.g., false positive rates in biopsy recommendations), which can obscure model shortcomings in specific clinical contexts. Tian et al. (104) introduced a comprehensive framework for evaluating medical AI applications, drawing upon the well-established Donabedian model of healthcare quality management and the DeLone & McLean model of information system success. Their approach underscores the necessity of incorporating clinical utility and subgroup-specific evaluations to ensure robust and context-sensitive performance assessment. The NHCKG model, as referenced in the literature, is designed to facilitate rapid and accurate medical evaluations, with a particular focus on constructing knowledge graphs tailored to disease staging. Myers et al. (105) explored the application of the image science framework in assessing medical imaging technologies, especially AI-integrated diagnostic tools. They highlighted that conventional metrics, such as AUC, may be insufficient for capturing the nuanced performance of AI systems in real-world clinical settings. They advocated for task-specific evaluation criteria (e.g., false positive rate analysis) and proposed that computational modeling and database development could help bridge existing knowledge gaps, thereby enabled a more holistic assessment of AI-driven medical imaging solutions.

### 5.3 Other limitations

#### 5.3.1 Ethical gaps

The ethical challenges associated with AI applications cannot be ignored. Issues such as data privacy, algorithmic bias, and health equity are urgent problems that need to be addressed. With the increasing collection and use of medical data, ensuring patient privacy rights and preventing data misuse have become key challenges that must be tackled. Additionally, potential biases in AI algorithms may lead to unfair treatment of certain groups, such as patients in low-resource environments, affecting the quality of their diagnosis and treatment. To promote the fair application of AI technology, it is essential to eliminate bias in the development and implementation of AI and ensure its accessibility in various social contexts. Moreover, the training of clinicians is crucial, especially in accurately diagnosing and making informed treatment decisions with the aid of AI. The capabilities and trust of doctors will directly impact the effectiveness of AI technology in clinical settings.

#### 5.3.2 Clinical translation and regulatory barriers

In the clinical translation of AI technology, especially when facing regulatory barriers such as the requirements of the US Food and Drug Administration and the EU CE certification, there are significant challenges. To promote the wide application of AI systems, these regulatory barriers must be overcome to enable AI enter clinical practices and be effectively validated. In this process, the collaborative model between clinicians and AI are crucial. Doctors not only need to understand the working principles of AI systems but also play a leading role in diagnostic and treatment decisions. Additionally, the application of AI in gynecological tumor diagnosis and treatment must ensure its cost-effectiveness, avoid excessive medical costs due to the introduction of technology and ensure its wide application is not restricted by economic pressure.

Therefore, although the research and development of AI in the field of gynecological oncology is advancing rapidly, many challenges still need to be overcome to achieve effective transformation from the laboratory to the clinic. Future research should focus on strengthening interdisciplinary cooperation, enhancing the training of clinicians, standardizing and regulating data processing procedures, promoting the wider application of AI technology in the early screening, precise diagnosis, treatment and prognosis prediction of gynecological tumors, and ensuring data privacy and ethical fairness.

## 6 Summary and outlook

The application of AI in the field of gynecological malignancies (CCA, EC and OC) has made remarkable progress, providing revolutionary tools for early screening and diagnosis, precise treatment and prognosis prediction. In terms of diagnosis, AI, through DL and ML technologies, has achieved automated analysis of medical images (such as ultrasound, MRI, CT) and pathological sections, with performance comparable to or even surpassing that of experts. For instance, AI has significantly reduced the false negative rate in CCA screening and demonstrated high sensitivity and specificity in OC image classification. In the treatment of gynecological malignancies, AI-assisted surgical navigation, radiotherapy target delineation and personalized treatment plan recommendation have significantly enhanced treatment accuracy and patients’ life quality. In prognosis prediction, AI has constructed high-precision recurrence risk models by integrating multi-omics data (such as genomics, radiomics and clinical indicators), provided a scientific basis for dynamic monitoring and precise intervention.

At present, many reviews focus on a single technology (such as imaging or genomic data) (106, 107) or on the application of a single cancer type (108, 109). These reviews lack a comprehensive discussion of multimodal data fusion. Komatsu et al. (110) explored the application of AI in oncology ultrasound imaging in their review, emphasizing the advantages of DL in image data analysis and pointing out that AI systems are superior to traditional imaging methods in diagnostic accuracy. Bhardwaj et al. (58) focused on traditional ML methods in their review but did not fully cover the rapidly developing DL technology. In contrast, this review hereby summarizes the latest AI technologies (such as multimodal learning and explainable AI) in gynecological oncology and discusses the clinical translation potential of these technologies. We not only summarizes the application of AI in single technologies (such as imaging analysis or genomics), but also particularly emphasizes the importance of multimodal data fusion. For example, in the diagnosis of OC, AI has constructed a high-precision prediction model by combining ultrasound images, clinical variables, and radiomics features. It covers the complete process (early screening, diagnosis, treatment, and prognosis prediction) of the three major gynecological malignancies, which is comprehensive and forward-looking, providing a more comprehensive perspective and helping researchers understand the comprehensive application of AI in gynecological oncology. In addition, some reviews may focus on technical details but lack discussions on clinical implementation challenges (such as data privacy, model generalization, and doctor-AI collaboration) (111). While we discuss in detail the challenges of AI in data standardization, ethical issues, and clinical translation, providing practical guidance for future AI research.

Looking ahead, the development of AI in the field of gynecological oncology will move towards greater intelligence, personalization, and standardization. Firstly, with the continuous optimization of algorithms and the accumulation of big data, the accuracy and interpretability of AI models will be further enhanced, enabling them to play a more significant role in clinical decision-making. Secondly, multi-center collaboration and the establishment of standardized datasets will help to address issues of data representativeness and annotation consistency, thereby strengthen the generalization ability of models. Additionally, the application of privacy protection technologies such as federated learning will make cross-institutional data sharing possible, further promotes the clinical transformation of AI. In terms of ethics and regulation, a unified evaluation framework needs to be established to ensure the fairness and safety of AI technology. Finally, the deepening of the AI-doctor collaboration model will redefine the diagnosis and treatment process of gynecological oncology, transforming from an “auxiliary tool” to an “intelligent partner”, ultimately improve the treatment outcomes and patients’ life quality.

In conclusion, AI technology is reshaping the screening, diagnostic, therapeutic and prognosis prediction paradigms in gynecological oncology. Despite challenges like data quality, algorithm interpretability, and clinical translation, with technological advancements and deeper interdisciplinary collaboration, AI is bound to play an irreplaceable role in the precision medicine of gynecological tumors, offering patients more efficient and personalized treatment experiences. Future research should continue to explore the potential of AI in multi-modal data integration, real-time dynamic monitoring, and clinical practicality, driving gynecological oncology towards new heights.
